# Fatty acid profile and in vitro biological properties of two Rosacea species (*Pyrus glabra* and *Pyrus syriaca*), grown as wild in Iran

**DOI:** 10.1002/fsn3.1352

**Published:** 2019-12-29

**Authors:** Saeid Hazrati, Mostafa Govahi, Saeed Mollaei

**Affiliations:** ^1^ Department of Agronomy Faculty of Agriculture Azarbaijan Shahid Madani University Tabriz Iran; ^2^ Department of Nano biotechnology Faculty of Biotechnology Amol University of Special Modern Technologies Amol Iran; ^3^ Phytochemical Laboratory Department of Chemistry Faculty of Sciences Azarbaijan Shahid Madani University Tabriz Iran

**Keywords:** antioxidant activities, fatty acids, *pyrus*, tocopherols

## Abstract

The high demands for the consumption of edible oils have caused scientists to struggle in assessing wild plants as a new source of seed oils. Therefore, in this study, the oil yield, fatty acid and tocopherol compositions, antioxidant and antibacterial activities of the oils obtained from Iran's two endemic plants (*Pyrus glabra* and *Pyrus syriaca*) were investigated. The obtained oil yields from the *P. glabra* and *P*. *syriaca* seeds were 33 ± 0.51 and 26 ± 0.28 w/w%, respectively. Oleic acid (C18:1) with the amount of 49.51 ± 1.05% was the major fatty acid in the *P. glabra* oil, while the main fatty acids in the *P. syriaca* seed oil belonged to linoleic acid (C18:2) and oleic acid (C18:1) with the amounts of 46.99 ± 0.37 and 41.43 ± 0.23%, respectively. The analysis of tocopherols was done by HPLC, and the results indicated that the *P. glabra* and *P. syriaca* seed oils were rich in α‐tocopherol (69.80 ± 1.91 and 45.50 ± 1.86 mg/100 g oil, respectively), constituting 86.24 and 89.01% of total detected tocopherols, respectively. The study on the reducing capacity of the oils indicated that the *P*.* glabra* oil had more reducing capacity than the *P. syriaca* oil. Moreover, the antioxidant activity of the *P. glabra* seed oil (43.4 ± 0.7 µg/ml) was higher than the *P. syriaca* seed oil (46.3 ± 1.2 µg/ml). Also, the investigation of the antibacterial activities indicated that the *P. glabra* and *P. syriaca* oils have an inhibitory effect on the studied bacteria. The results indicate that the oils of these plants can be appropriate sources of plant oils which can act as natural antibacterial agents.

## Graphical abstract

One of the most important findings of this study is the introduction of *Pyrus glabra* and *Pyrus syriaca* as a new source of plant oils. The significant amount of antioxidants, especially in *P. glabra* oil, may serve as a dietary source of natural antioxidants and increase the stability and quality of food products. The results showed that the *P. glabra* seed oil is rich in α‐tocopherol. Hence, the potential utilization of these seed oils as a raw material for food, chemical, cosmetic, and pharmaceutical industries could be favorable.

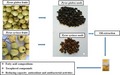

## 1 INTRODUCTION

Iran is a country with a specific geographic location containing various climates with endemic valuable medicinal plants, and the study on the endemic plants producing bioactive secondary metabolites is always an interesting field (Mozaffarian,^2012^). It is important to introduce new sources of the oils or utilize agro‐industrial by‐products as a source of oils, which can be good sources not only essential fatty acids but also of minor bioactive compounds such as tocopherols, sterols, carotenoids (Górnaś, 2015; Górnaś, Mišina, Lāce, Lācis, & Segliņa, 2015; Górnaś & Rudzińska, 2016; Górnaś, Siger, & Segliņa, 2013; Górnaś, Soliven, & Segliņa, 2015; Hazrati, Nicola, Khurizadeh, Alirezalu, & Mohammadi, 2019). These oils could improve the human health and reduce the chronic diseases such as hyperlipidemia, arrhythmia, rheumatoid arthritis, cancer, inflammatory, and autoimmune (Dhouioui et al., 2016; Ramadan, 2019).

Anchochek (*Pyrus glabra*) and Hermo (*Pyrus syriaca*), wild pears, are perennial plants belonging to *Rosaceae* family (Jalilian, Zarei, & Erfani‐Moghadam, 2018). *P. glabra* and *P. syriaca* are endemic to Iran's southwest regions (Sepidan region), which are 6 meters in height with smooth and gray leaves, and sometimes their branches appear prickly (Zamani, Riasat, Saadat, & Hatami, 2009). These trees have oblong and lanceolate leaves with small and white flowers with the green fruit which turns to brownish to black color after ripening. The fruit is spherical in shape with diameter of 2.5 cm and contains about 6 large seeds with the weight of one thousand seeds of 120 g (Hazrati Yadekori, Alirezalu, Tahmasebi‐Sarvestani, & Alirezalu, 2012; Sharifani, Kimura, Yamamoto, & Nishtani, 2017). Their fruits ripen in August up to September, and after gathering of the fruits, the seeds with black color are separated (Hashemi et al., 2018). From ancient times, the oils existing in the seeds of these plants have been used for body strengthening. Their oils have traditionally been utilized as a diuretic (Hashemi et al., 2018; Hazrati Yadekori et al., 2012). The pear fruits, like many others of Rosaceae family, contain an exceptionally small quantity of seeds (about ten tiny seeds per fruit) which can furnish 15%–31% oleaginous attributes. The profile of bioactive compounds in the oils is affected by species and variety (genotype) of the Rosaceae family (Górnaś, Mišina, Ruisa, et al., 2015; Górnaś, Rudzińska, et al., 2016; Rudzińska, Górnaś, Raczyk, & Soliven, 2017). The most fascinating oleaginous compounds in different species of the pear seed oil include unsaturated fatty acids, tocochromanols, and phytosterols. Out of the pear seed oil fatty acids, linoleic acid (C18:2) level was found to be higher than most of the frequently used edible oils (Mushtaq, Akram, Ishaq, & Adnan,^2019^). Other recognized fatty acids in the seeds of pear species can be cited as palmitoleic acid, palmitic acid, stearic acid, and alpha‐linolenic acid (Mushtaq et al.,^2019^). According to the research done by Hashemi et al. (2018), the oil content of *P. glabra* was 22.40% and linoleic and oleic acids were reported as its major existing fatty acids.

The use of antioxidants is very useful for the strengthening of body antioxidant defense system in order to control, prevent, or treat diseases. The tendency toward natural antioxidants has been increased due to the side effects of synthetic ones (Kurutas, 2016). The natural antioxidants are found in different parts of the plants such as root, stem, leaf, flower, and fruit. These compounds, because of their presence with other compounds in plants, have biological balance, so they are not accumulated in the body and cause the minimum side effects (Memvanga, Tona, Mesia, Lusakibanza, & Cimanga, 2015).

The bacterial resistance against the majority of antibiotics has provided further attention, which led to the search and introduction of new antimicrobial compounds with plant origin. Some herbal compounds that have plentiful applications in the food industry are plants, in which some of these oils exhibit antimicrobial properties. The antimicrobial properties of some seed oils have been reported previously (Dhouioui et al., 2016; Karimi, Jaafar, Ghasemzadeh, & Ebrahimi, 2015).

*P. glabra* and *P. syriaca* have spread in the southwest of Iran and have been consumed as nuts. Hence, the objective of this research is to compare the oil yield, the composition of fatty acids and tocopherols, antioxidant and antibacterial activities in the oil of two *Pyrus* seeds, which makes possible the determination of the species with the maximum quantity of fatty acid and the highest biological activity.

## 2 MATERIALS AND METHODS

### 2.1 Seed collection

*Pyrus glabra* and *Pyrus syriaca* fruits were collected from the natural forest north of Sepidan, Fars Province (southwest of Iran) in September of 2018. The GPS location details were the longitude of 40ʹ51°50ʹ51°E, latitude of 20ʹ30°30ʹ30°N, and altitude of 2,890 m.

The sampling was done by a randomized collection of 10–15 trees in an area of about 5,000 m^2^. Matured fruits were isolated manually from the aerial parts in our laboratory to obtain a weight of 1,000–1,200 g of each sample. The seeds were removed from the fruit by hand. The seeds were washed with sterile water and then dried at room temperature (25 ± 3°C) for 10 days before storing in a sealed container at 4°C until oil extraction (for a maximum of 15 days).

### 2.2 Extraction and measurement of oil content

The seeds were milled using a grinder (Naniwa‐97, Iran) to obtain a fine powder, and then, 300 ml of n‐hexane was added to 30‐g dried powdered seeds and placed in the Soxhlet extractor. After 5 hr, the solvent was removed with the rotary evaporator and the oil percentage was calculated.

### 2.3 Preparation of methyl ester oil

Five millilitre of sodium hydroxide (2 v/w %) was added to 0.05 g of the obtained oil and heated for 10 min. Then, 2 ml of boron trifluoride was added, and the reflex action was continued for 3 min. Later, 1.5 ml of hexane was added in the sample and shaken for 5 min. Finally, the hexane phase was separated and the dehydration of the oil was done using sodium sulfate and injected into the GC device (Metcolf, Schmitz, & Pelka, 1966).

### 2.4 Analysis of fatty acid methyl ester by gas chromatography

Fatty acid methyl ester was analyzed using a Varian gas chromatograph (model 6890) equipped with a BPX70 column (30 m × 0.22 mm, 0.25 µm film thickness). The oven temperature program was started by an increase from 158°C to 210°C by a ramp of 2°C/min and was subsequently kept consistent at 210°C for 20 min. The injector temperature and detector temperature were adjusted at 230°C, and 240°C, respectively, and the flow of carrier gas (helium) was 1.2 ml/min. The injection into the GC device was done by the split method in the ratio of 1:100 (Azadmard‐Damirchi & Dutta, 2008).

### 2.5 Analysis of tocopherol compositions

The analysis of tocopherols was carried out using a Knauer HPLC instrument (Berlin, Germany) equipped with a fluorescence detector, a pump, and a Rheodyne 7,125 injector. Chromatography was performed on a NH_2_ column (250 × 4.6 mm i.d., 5 μm) using hexane‐isopropanol (99:1, v/v) as the mobile phase. The flow rate, column temperature, and wavelength of the detector were adjusted at 1 ml/min, 25°C, and 290 and 330 nm, respectively.

### 2.6 The Folin–Ciocalteu reagent (FCR) assay

The reducing capacity of the oils was determined by the use of the Folin–Ciocalteu colorimetric method. For the extraction of reducing compounds from the oil, 10 ml of water: methanol (30:70 v/v%) was added to 2 ml of each oil, followed by vortex for 60 s, and then centrifuged for 5 min at 3,000 rpm. The extraction was done three times. All the obtained extracts were combined and then concentrated until dryness. After the extraction process, 2 mg of the extract was redissolved in methanol and then mixed with 500 µl of FCR (10 v/v %). After 5 min, 500 µl of 7% carbonate sodium was added into the mixture and the absorbance of the samples (after keeping in the dark for 2 hr) was measured at the wavelength of 765 nm. Thus, the FCR reducing capacity was expressed as gallic acid equivalents (µM) (Huang, Ou, & Prior, 2005).

### 2.7 Measurement of free radical scavenging activity (DPPH assay)

The antioxidant activity of the oil was estimated according to the method reported by Mollaei, Sedighi, Habibi, Hazrati, and Asgharian (2019) with some modifications. Briefly, 150 µl of the DPPH solution (0.1 mg/ml in methanol) was mixed with 150 µl of the oil with different concentrations (250, 125, 62.5, 31.2, 15.6, and 7.8 µg/ml in methanol). The mixture was incubated at 25°C for 30 min, and then, the sample absorbance was measured at 517 nm. The percentage of radical scavenging activity was calculated according to the following equation:

$$\text{Radical scavenging activity}\left(\%\right)=\frac{A_{\text{control}}-A_{\text{sample}}}{A_{\text{control}}}\times 100.$$

### 2.8 Antimicrobial activities

The antimicrobial activity of the oils was tested individually against a range of seven microorganisms, including *Bacillus pumilus* (PTCC 1274), *Bacillus subtilis* (ATCC 465), *Escherichia coli* (ATCC 25922), *Staphylococcus aureus* (ATCC 25923), *Klebsiella pneumoniae* (ATCC 10031), *Bacillus cereus* (PTCC 1015), and *Saccharomyces cerevisiae* (ATCC 9763). It was determined by the disk diffusion method using Mueller–Hinton agar plates (Eftekhar, Raei, Yousefzadi, Ebrahimi, & Hadian, 2009), with the determination of inhibition zones. Also, the MIC values were determined by the broth microdilution assay. All experiments were done in triplicate.

### 2.9 Statistical analysis

All data were analyzed by SAS software (SAS 9.2). One‐way ANOVA was carried out, and significant differences between groups were measured by Duncan's multiple range test at *p* < .05.

## 3 RESULTS AND DISCUSSION

### 3.1 Oil content and fatty acid compositions

The results indicated that the obtained yields for oil extraction from the seeds of *P. glabra* and *P. syriaca* were 33.0 ± 0.5 (v/w %) and 26.0 ± 0.3 (v/w %), respectively (Table 1). Yukui, Wenya, Rashid, and Qing (2009) extracted lipids from the seeds of *P. communis* at a relatively higher temperature applying petroleum ether as an extraction solvent and observed that the oil yield was 17.9%. Górnaś, Rudzińska, et al. (2016) extracted the oils from the seeds of eight pear (*P. communis* L.) cultivars using vortex and ultrasonic as an extraction method. They concluded that the oil yield in pear seeds ranged between 16.3 and 31.5 (w/w %). Hashemi et al. (2018) reported the oil content of 22.40% for the seeds of *P. glabra*. Also, in another study, the oil content was stated between 12.1 and 29.9 (w/w %) in the seeds of different apple cultivars (Fromm, Bayha, Carle, & Kammerer, 2012). Our results indicated that the oil extraction yields were higher compared to the other Rosaceae family plants such as *Rosa canina* (8%–11%) and pear (17%), the source for the production of plant/vegetable oil (Saeedi & Omidbaigi, 2009; Yukui et al., 2009). Overall, our results and the above‐mentioned studies showed that the oil yield in the seeds of pear fruit could vary with agro‐climate conditions, fruit cultivar, and extraction solvent or method. Moreover, due to the concern of consumer health, the presence of amygdalin (cyanogenic glycoside) in the products obtained from the seeds of the Rosaceae family should be checked, as recommended previously (Górnaś, Mišina, Olšteine, et al., 2015; Makarova et al., 2015; Senica, Stampar, Veberic, & Mikulic‐Petkovsek, 2017).

**Table 1:** Comparison of the yield, reducing capacity, and antioxidant activity (IC_50_) of two *Pyrus* species

Sample	Oil yield (v/w %)	Reducing capacity (µM)	IC_50_ (µg/ml)	BHT
*Pyrus syriaca*	26.0 ± 0.3	28.7 ± 1.2	46.3 ± 1.2	23 ± 0.2
*Pyrus glabra*	33.0 ± 0.5	39.8 ± 2.1	43.4 ± 0.7	

Note

Mean value ± standard error (*n* = 3).

Abbreviation: BHT, Butylated hydroxytoluene.

Based on the results of both species’ fatty acid analysis, eight fatty acids were identified (Table 2). Linoleic acid (46.99 ± 0.37%) and oleic acid (41.43 ± 0.23%) were identified as the main fatty acids in the *P. syriaca* seed oil, while in the *P*. *glabra* seed oil, their percentage was 49.51 ± 1.05 and 37.47 ± 0.36%, respectively. Also, *P*.* glabra* and *P. syriaca* seed oils had a considerable amount of palmitic acid of 7.89 ± 0.04 and 8.75 ± 0.02%, respectively. Other fatty acids in the *P*.* glabra* seed oil included palmitoleic acid, stearic acid, linoleic acid, alpha‐linolenic acid, arachidic acid, methyl ester heneicosanoic acid, and in the *P. syriaca* seed oil included palmitic acid, palmitoleic acid, stearic acid, alpha‐linolenic acid, arachidic acid, and gondoic acid. Study on the seed oil of eight *Pyrus communis* cultivars indicated that the oil yield ranged between 16.3 and 31.5 (w/w%) and the main fatty acids were linoleic acid, oleic acid, and palmitic acid, all three representing 96%–99% of the total detected fatty acids (Górnaś, Rudzińska, et al., 2016). In another research, eleven fatty acids were detected in the *Pyrus* seed oil, in which the dominant fatty acids were oleic acid (56.80 g/100 g oil), stearic acid (20.28 g/100 g oil), and palmitic acid (6.39 g/100 g oil) (Yukui et al., 2009). According to the research done by Hashemi et al. (2018), the oil content of *P. glabra* was 22.40% with the linoleic and oleic acids as the major existing fatty acids. Our results were similar to those of the above‐mentioned studies that demonstrated that stearic acid, palmitic acid, linoleic acid, and oleic acid were the major fatty acids in different species of *Pyrus* seeds, while other fatty acids such as linoleic acid, palmitoleic acid, behenic acid, arachidic acid, and gondoic acid were not found in all the *Pyrus* seeds.

**Table 2:** Fatty acid composition of oils obtained from two *Pyrus* species

Common name	Symbol	% of total fatty acids	
		*Pyrus glabra*	*Pyrus syriaca*
Palmitic acid	C16:0	7.89 ± 0.25	8.757 ± 0.23
Palmitoleic acid	C16:1	0.23 ± 0.06	0.191 ± 0.01
Stearic acid	C18:0	2.76 ± 0.69	1.25 ± 0.05
Oleic acid	C18:1	49.51 ± 1.05	41.43 ± 0.23
Linoleic acid	C18:2	37.47 ± 0.36	46.99 ± 0.37
α‐Linolenic acid	C18:3	0.19 ± 0.05	0.17 ± 0.01
Arachidic acid	C20:0	1.32 ± 0.33	0.65 ± 0.2
Gondoic acid	C20:1	—	0.55 ± 0.01
Heneicosanoic acid methyl ester	C21:0	0.61 ± 0.15	—
ΣSFA	—	12.58	10.66
ΣMUFA	—	49.74	42.17
ΣPUFA	—	37.94	47.16
ΣMUFA/ΣPUFA	—	1.31	0.89

Note

Mean value ± standard error (*n* = 3).

Abbrevitions: MUFA, monounsaturated fatty acids; PUFA, polyunsaturated fatty acids; SFA, saturated fatty acids.

The total unsaturated fatty acids in the *P*.* glabra* and *P. syriaca* oils were 87.68 and 89.33%, and the content of saturated fatty acids was 12.58% and 10.66%, respectively. The saturated fatty acids that occurred in the highest amount were palmitic acid and stearic acid, while the unsaturated fatty acids were linoleic acid and oleic acid in the seeds. These findings were in agreement with data reported by other authors (Górnaś, Rudzińska, et al., 2016; Hashemi et al., 2018; Yukui et al., 2009) who observed that unsaturated fatty acids were the most abundant fatty acids in pear seed oils. Górnaś, Rudzińska, et al. (2016) investigated the chemical composition of seed oils extracted from different *P. communis* cultivars. They observed that the ratio of unsaturated to saturated fatty acids in pear seed oils was in the range of 8.32–11.35. In another research, the main fatty acids were the unsaturated fatty acids (around 85%), including the monounsaturated fatty acids and the polyunsaturated fatty acids (Hashemi et al., 2018).

The results showed that the amount of linoleic acid in *P. syriaca* seed oil with 46.99 ± 0.37% was higher than *P*.* glabra*, with a value of 37.47 ± 9.36%. The ratio of monounsaturated fatty acids to polyunsaturated fatty acids was calculated by 0.89 and 1.31 in the *P*.* glabra* and *P. syriaca* seeds, respectively, as an indicator for the tendency of autoxidation. This ratio has been reported in the *Pistacia khinjuk* fruits (2.89) (Asnaashari, Hashemi, Mahdavian Mehr, & Asadi Yousefabad, 2015), *Trichodesma indicum* (0.43) (Górnaś et al., 2019), and *P*.* glabra* (0.89) (Hashemi et al., 2018). Górnaś, Rudzińska, et al. (2016) showed that the overall percentage of saturated, monounsaturated, and polyunsaturated fatty acids in the pear seed oil was 9.48%, 31.21%, and 59.32%, respectively. Moreover, it has been shown that the oil compounds in the seeds of pear species fruit may vary with agro‐climate conditions and fruit cultivar (Mushtaq et al.,^2019^).

### 3.2 Tocopherol composition

The tocopherol compounds of *P. glabra* and *P. syriaca* oils are presented in Table 3. As it is shown, the total amount of tocopherols in *P*.* glabra* oil (69.80 ± 1.91 mg/100 g oil) was higher than *P. syriaca* oil (45.50 ± 1.86 mg/100 g oil). The *P*.* glabra* and *P. syriaca* seed oils show high concentration of total tocopherols, and this concert is higher than what has been obtained in edible oils like *Helianthus annuus* (44.0 mg/100 g), *Sesamum indicum* (33.0 mg/100 g), *Arachis hypogaea* (17.0 mg/100 g), *Vitis vinifera* (24–41 mg/100 g), *Elaeis guineensis* (26 mg/100 g), and *Carthamus tinctorius* (24–67 mg/100 g), according to the Codex Alimentarius Commission (1999) and Codex Alimentarius Commission (2009). According to some reports, the amount of tocopherol obtained in our experiment was lower in comparison with other pears (Górnaś, Mišina, Lāce, et al., 2015; Górnaś, Rudzińska, et al., 2016). Four tocopherols were detected and quantified in the samples. According to the results, α‐tocopherol was the main tocopherol in the *P*.* glabra* and *P. syriaca* oils (60.2 and 40.5 mg/100 g), respectively. Hashemi et al. (2018) showed that the total tocopherol content in the *P. glabra* was much lower than that reported by Górnaś, Rudzińska, et al. (2016) in the *P. communis* seed oils. Also, the amounts of a, ß, γ, and δ tocopherols in the *P. glabra* and *P. syriaca* seed oils were different in previous researches. Górnaś & Rudzińska, et al. (2016) reported the γ‐tocopherol as the main tocopherol, while Hashemi et al. (2018) observed *P. glabra* as the richest source of α‐tocopherol. These researchers concluded that the *P. glabra* seed oil has the highest amount of α‐ tocopherols and other tocopherols (ß, γ, and δ) were found in traces, in which our results confirm these observations too. Tocopherols are produced in seeds in variable levels, and antioxidative activity varies between individual compounds. Tocopherols persuade a protective effect against oxidative stress related to metabolic syndrome and are also essential for regular neurological function (Aggarwal, Sundaram, Prasad, & Kannappan, 2010).

**Table 3:** Vitamin E (tocopherols) contents of oils of two *Pyrus* species

Compounds	Concentration (mg/100 g oil)	
	*Pyrus glabra*	*Pyrus syriaca*
α‐Tocopherol	60.2 ± 1.18	40.50 ± 1.41
β‐Tocopherol	4.1 ± 0.40	3.10 ± 0.17
γ‐Tocopherol	2.3 ± 0.14	1.10 ± 0.19
δ‐Tocopherol	3.2 ± 0.19	0.80 ± 0.09
Total of tocopherols	69.80 ± 1.91	45.50 ± 1.86

Note

Mean value ± standard error (*n* = 3).

### 3.3 Antioxidant activity (DPPH assay) and reducing capacity of the oils

The results of antioxidant activity indicated that the oil inhibitory activities depend on the concentration of the oil and the increase in the concentration increased the inhibitory percentage. The values of IC_50_ in the *P*.* glabra* and *P. syriaca* oils were 43.4 ± 0.7 and 46.3 ± 1.2 μg/ml, respectively (Table 1). Based on the results, the *P*.* glabra* oil has the higher antioxidant activity than the *P. syriaca* oil. To our knowledge, there are no studies done on the antioxidant activity of *Pyrus* seed oils. Therefore, the obtained results were compared with other plants of the Rosaceae family. Simirgiotis, Quispe, Bórquez, Arce, and Sepúlveda (2016) concluded that the small fruits of *P. communis* had a higher antioxidant activity (8.61 ± 0.65 μg/ml) and this activity can be related to the presence of several phenolic compounds. The antioxidant activity of the pear cultivars indicated that the potent antioxidant activity was detected in pear extracts of the “Grabova” cultivar (Liaudanskas, Zymonė, Viškelis, Klevinskas, & Janulis, 2017). A similar report has been issued as to the antioxidant activity of *Pyrus* species (Shan, Huang, Shah, & Abbasi, 2019).

The FCR was used to assess the reducing capacity of the seed oils (Górnaś, Dwiecki, et al., 2016; Górnaś, Šnē, Siger, & Segliņa, 2014). The results showed that the maximum reducing capacity belonged to the *P*.* glabra* seed oil with the quantity of 39.8 ± 2.1 µM, and *P. syriaca* oil contained 28.7 ± 1.2 µM.

In the *P*.* glabra* seed oil, the observed higher antioxidant activity compared with the *P. syriaca* seed oil could be associated with higher amounts of reducing compounds and tocopherols. These compounds may have hydroxyl groups on the aromatic ring, and phenolic proton dissociation leads to a phenolate anion, which is capable of reducing FCR (Box, 1983). This supports the notion that the reaction occurs through an electron transfer mechanism. So, antioxidant compounds have a significant impact on the reduction of free radicals and on the prevention of hydroxyl conversion to free radicals, which is the principal factor in the creation of cancer in humans. Varela (2016) demonstrated that the mechanism phenolic compounds for antioxidant activities are due to the reduction of free radicals such as fat peroxides, anions, superoxides, and hydroxide radicals. Nimse and Pal (2015) represented that antioxidant compounds are capable of trapping single oxygen as well. The acquired results from measuring reducing capacity revealed that the *P*.* glabra* oil may have the maximum amount of antioxidant compounds. The antioxidant compounds in the oils perhaps act as the reduction factor and, through electron release, react with radical compounds and convert them into resistant compounds, which eventually result in the neutralization of free radical chain (Leopoldini, Marino, & Russo, 2004). Hence, the great antioxidant properties of *P*.* glabra* plant oil can be attributed to its higher reducing capacity of the oil. So through the consumption of oils with further antioxidant properties such as *P*.* glabra* oil, it is feasible to partially decrease the destructive effects of free radicals in the body.

### 3.4 Antibacterial activity

No research has been conducted as to the antimicrobial activity of the *P. glabra* and *P. syriaca* oils in Iran and other countries so far. However, some experiments have been accomplished about the antibacterial effect of other seed oils and reveal the antibacterial activity of them (Dhouioui et al., 2016; Huang, Xue, He, & Zhao, 2019; Karimi et al., 2015; Shukla et al., 2018). The antibacterial activity of the *P. glabra* and *P. syriaca* oils was tested against five gram‐positive and two gram‐negative bacteria. The results, according to the disk diffusion method and minimum inhibitory concentration (MIC) values showed that the oils indicated moderate‐to‐high inhibitory activity against the tested bacteria (Tables 4 and 5). The results suggested that *Bacillus cereus* with MIC of 7.5 mg/ml is the most susceptible bacterium against the *P*.* glabra* oil.

**Table 4:** In vitro antimicrobial activities^a^ of the oils obtained from two *Pyrus* species (disk diffusion method) against various microorganisms

Sample	Microorganism						
	*B. pumilus*	*B. subtilis*	*S. aureus*	*B. cereus*	*K. pneumonia*	*E. coli*	*S. epidermidis*
*Pyrus glabra*	12	14	12	12	10	10	12
*Pyrus syriaca*	12	12	10	11	12	11	10
Ampicillin^b^	15	14	13	nt	Nt	12	19

a Zone of inhibition (in mm) includes diameter of the disk (6 mm), values as mg/ml, (–): inactive, (7–13): moderately active, (>14): highly active, nt: not tested, a quantity of 10 µl of EtOH without sample (negative control) was inactive.

b Tested at 10 μg/disk. All experiments were done in triplicate.

**Table 5:** Minimum inhibitory concentration (MIC [mg/ml]) of the oils obtained from two *Pyrus* species

Sample	Microorganism						
	*B. pumilus*	*B. subtilis*	*S. aureus*	*B. cereus*	*K. pneumoniae*	*E. coli*	*S. epidermidis*
*Pyrus glabra*	15	15	>15	7.5	>15	>15	15
*Pyrus syriaca*	15	15	15	15	>15	15	>15
Ampicillin^a^	15	15	15	nt	nt	15	15

Abbreviation: nt, not tested.

a Tested at 10 μg/disk. All experiments were done in triplicate.

## 4 CONCLUSION

The results obtained from the present study indicated that the oil extraction yield of *P glabra* and *P. syriaca* seeds was 33.00 ± 0.51 and 26.00 ± 0.28% v/w, respectively. Also, the obtained oils contained a high source of unsaturated fatty acids, and in both of them, oleic acid and linoleic acid were found in the highest amount. In addition, significant differences were observed between vitamin E (tocopherols) contents of the oils and the highest level of them belonged to the *P. glabra* oils. Furthermore, the oils obtained from the seeds of mature fruit have appropriate antioxidant activity as well as considered as a suitable source of moderate antibacterial properties.

## CONFLICT OF INTEREST

The authors declare that they do not have any conflict of interest.

## ETHICAL APPROVAL

This research does not include any human and animal testing.

## INFORMED CONSENT

Written informed consent was obtained from all study participants.
